# The Texas Quarantine Law, and Other Matters

**Published:** 1899-10

**Authors:** 


### THE TEXAS QUARANTINE LAW, AND OTHER MATTERS.

We reproduce in this issue the quarantine law now in force, the only law relating in any way to the public health that is on the Texas statutes and in force. It will be seen that it relates solely to quarantine as against "contagious and infectious diseases," and makes no allusion to any other feature of sanitation. It is doubtless a good law, so far as it goes, or rather, would be, if it were strictly carried out. Dr. Swearingen, who was the author of the law, was never able to secure the cordial co-operation either of the judges, whose duty it is to appoint the county physicians, or of the county physicians when appointed. The law requires the judge in each county, after each election, to appoint a county physician, and the duties of the latter are set forth in the act, so far as relates to quarantine; but as there is no penalty for neglect of the duty by the judge, the appointments, in the majority of cases, are not, and have not been made. The present incumbent of the State "Health" office, Dr. Blunt, has touched the judges up, and has secured a pretty fair list of county physicians.

I have said that the State Health Officer heretofore has not had the co-operation of county physicians except to a limited extent, and only when and where an outbreak of some quarantinable disease has occurred. The county physicians for the most part do not seem to properly understand what is expected of them, nor realize that they are a part of the State machinery for the protection of the people from epidemic diseases.

As it seems to be impossible to secure further legislation in the direction of increased sanitary methods,—the medical profession of the State having signally failed in every effort to arouse an interest in the subject on the part of either governor or legislator,—and those efforts have been made every session of the Legislature for the past twenty years,—it behooves the medical men of the State to try to make the most of the law above referred to. If every county physician would take a wider view of his duties and responsibilities than can be inferred from the letter of the law, and bring to bear upon the subject zeal and intelligence, he can accomplish much more than is expected of him in the most conscientious and intelligent discharge of the duties stipulated in the act. Because there is no State law requiring sanitary measures looking to the prevention of indiginous diseases, there is no reason why such measures should not be instituted by county and municipal authorities; and every county and city physician should personally enlighten those authorities upon the subject, and upon the necessity of such measures. There is no reason why a county commissioners' court should not make ordinances with penalty attached for violation thereof to prevent the pollution of streams, springs, wells, etc.; to compel the ditching of land where water stands, and provide for drainage in all such places; to compel the removal and destruction of all excreta, the abolition of all sinks or underground privies, and where surface privies are used, to compel their cleansing at stated intervals; and many other details that it would seem unnecessary to state. Because there is no State law to require vaccination and revaccination, there is no reason why there should not be such a law in every county and town. What would be tantamount to compulsory vaccination would be an ordinance in every town and by every commissioners' court forbidding the admission into any school of any child who cannot show evidence (a certificate from a reputable physician) of successful vaccination, making it a finable offense on the part of the teacher or superintendent for violation of the act; and forbidding the employment by any person or persons of any laborer or help in any capacity, without a certificate of successful vaccination. Let the physicians of Texas become aroused to a realization of the lamentable fact that they can expect nothing in the way of legislation on this subject, and let them take up the question at home. Not only those who have been appointed "county physician," but all good and intelligent physicians should take an interest in this matter and cause to be instituted at home the measures best calculated to prevent typhoid fever, diphtheria, dysentery, consumption, etc., diseases that may and do originate in our midst. A campaign of sanitary education of the masses should be inaugurated. The Sanitary Plumbers' League have started such campaign, and Mr. Hyde is doing good work. They should have the cordial support of the medical profession. The medical journals are read only by medical men, and the gospel of sanitation there preached never reaches the people. Individual physicians should disseminate it by word of mouth, and through local papers. San Antonio has set the pace; let all up-to-date doctors follow it.

